# Mediterranean Alcohol-Drinking Pattern and Alcohol-Related Cancer Incidence in the “Seguimiento Universidad de Navarra” (SUN) Cohort

**DOI:** 10.3390/medsci14010020

**Published:** 2025-12-31

**Authors:** María Barbería-Latasa, Estefanía Toledo, Maira Bes-Rastrollo, María Olmedo, Rafael Pérez-Araluce, Alfredo Gea, Miguel Ángel Martínez-González

**Affiliations:** 1 Department of Preventive Medicine and Public Health, University of Navarra, 31008 Pamplona, Spain; mbarberia.3@unav.es (M.B.-L.); etoledo@unav.es (E.T.); mbes@unav.es (M.B.-R.); molmedo@unav.es (M.O.); ageas@unav.es (A.G.); 2 Instituto de Investigación Sanitaria de Navarra (IdiSNA), Navarra Institute for Health Research, 31008 Pamplona, Spain; 3 Biomedical Research Network Center for Pathophysiology of Obesity and Nutrition (CIBEROBN), Carlos III Health Institute, 28029 Madrid, Spain

**Keywords:** Mediterranean diet, Mediterranean alcohol-drinking pattern, cancer, wine, moderate drinking, Cohort

## Abstract

**Background/Objectives**: Since 1988, the IARC has classified alcohol as a type 1 carcinogen, causally linked to seven types of cancer (oral cavity, pharynx, larynx, esophagus, colorectum, liver and breast carcinomas). Several agencies, such as the WHO and the IARC, hold that there is a direct monotonic association between any gram of alcohol consumed and the risk of cancer, regardless of the drinking pattern. On the other hand, an expanding body of evidence indicates that drinking pattern may substantially modify the effect of alcohol consumption. The Mediterranean alcohol-drinking pattern (MADP) includes different aspects of alcohol consumption, such as preference for red wine, moderate alcohol consumption with meals, spreading consumption over the week and avoiding binge drinking. Conformity to this pattern has shown inverse associations with all-cause mortality, cardiovascular disease and diabetes. However, its relationship with cancer incidence has not been studied yet. Our objective was to assess how alcohol consumption patterns, with particular emphasis on the MADP, relate to the incidence of the seven alcohol-related cancers. This information is needed to support cancer prevention recommendations that may go beyond the amount of alcohol consumed to also include the drinking pattern. **Methods**: We prospectively followed 19,541 participants in the SUN (“Seguimiento Universidad de Navarra”) cohort for a median of 13.8 years. We classified participants into four groups, namely, abstainers and three further groups according to their adherence to the MADP score (low, moderate and high). **Results**: A substantial reduction in the risk of alcohol-related cancer incidence was observed only in men for high versus low adherence to the MADP, with an adjusted hazard ratio (HR) of 0.44 (95% confidence intervals (CIs) (0.21–0.92)). The category of moderate adherence to the MADP showed a lower risk of cancer incidence with a tendency towards statistical significance (HR = 0.56, 95% CI, 0.30–1.06). For women, no result reached statistical significance. **Conclusions**: Based on the available evidence, separate messages by sex should be delivered. In men, the association between alcohol and cancer goes beyond the amount of alcohol consumed, and a Mediterranean drinking pattern may be beneficial even for alcohol-related cancers. Men should, therefore, receive an additional message: among alcohol consumers, greater adherence to the MADP may help lower their risk of developing alcohol-related cancers. No benefit is supported for the MADP against alcohol-related cancers in women.

## 1. Introduction

Alcohol is widely consumed worldwide and is associated with a wide range of diseases and injuries. While consumption has declined in recent years, in regions such as the Americas, Europe and the Western Pacific, more than half of the population consumes alcohol [1]. This makes alcohol a key issue for global public health.

In 1988, the International Agency for Research on Cancer (IARC) classified alcohol as a group 1 carcinogen, the most severe category. Cancer is now a leading risk factor for the global burden of disease, and it is estimated that 4.1% of new cancer cases in 2020 were attributable to alcohol consumption [2,3]. The IARC classifies seven types of cancer causally related to alcohol consumption: oral cavity, pharynx, larynx, esophagus (squamous cell carcinoma), colorectum, liver (hepatocellular carcinoma) and breast carcinoma. The impact of alcohol consumption is not the same for all cancers, with oesophageal, liver and breast cancers showing the highest number of alcohol-related cases [3]. Alcohol is also associated with an increase in recurrence after diagnosis [4,5,6]. On the other hand, interaction with smoking has been frequently reported, with associations greater than their multiplication [3]. Tobacco smoking is, therefore, considered an important effect modifier.

The available evidence suggests a linear effect of alcohol on cancer, with significantly increased risks for mouth, pharynx, esophagus and breast cancers, at any level of alcohol consumption. However, for the risk of colorectal or liver cancer, intakes above 30 or 40 g/day [7] are required to observe an increased risk. Nevertheless, there are also other studies which reported a J-shaped association, with a lower risk for light drinkers as compared to non-drinkers [8,9]. Similarly, there appears to be an inverse association with alcohol consumption for kidney cancer [10,11] and non-Hodgkin’s lymphoma [12,13]. As a result of this controversy, several studies have been carried out to provide further evidence of the link between alcohol intake and cancer but with mixed results. The latest global burden of disease (GBD) 2020 (published in 2022) estimated small overall health benefits for different levels (always low to moderate) of alcohol consumption according to age, sex and geographical distribution [14], in contrast to the previous GBD, which recommended zero alcohol consumption for everybody [15]. However, there is enough evidence to support that high levels of alcohol consumption are harmful for all sectors of the population. Nevertheless, light or moderate drinking might be associated with lower [8,9] or statistically non-significant [16,17,18] differences in cancer risks in several studies. With the available evidence, the benefits attributed to alcohol on diabetes, cardiovascular disease and mortality must be weighed individually against other risks, including cancer risks [17].

Other aspects of the association between alcohol and cancer have been analyzed, such as the influence of the type of beverage and the frequency of consumption. Agencies such as the IARC, American Cancer Association, National Institutes of Health (NIH) and World Cancer Research Fund (WCRF) argue that it is not the type of beverage that influences the association with cancer but the ethanol content and the frequency of consumption. Regarding the pattern of consumption, a concentrated pattern, i.e., binge drinking, has been associated with a significantly increased risk of cancer [19,20]. In contrast, other healthier drinking patterns, such as the Mediterranean alcohol-drinking pattern (MADP), have shown statistically significant results for reduced risk of mortality [21,22], cardiovascular disease (CVD) [23] and diabetes [24]. This pattern is characterized by moderate consumption of red wine with meals, consumption spread over the week and avoidance of binge drinking. Consideration of not only the type of beverage but also the drinking pattern, may have a greater influence on cancer. However, there are currently no studies on MADP and cancer. Some of the points included in the operational definition of the drinking pattern score have been studied, such as moderate drinking [25,26,27], red wine consumption [7,27,28,29], drinking with meals [18,30] or binge drinking [19,20], but no research has evaluated all of them in a single comprehensive score. Biologically, the effect of alcohol on cancer is likely to be lower when following the MADP due to the washout effect of food [30], the polyphenol content of wine [31,32] and the moderate consumption spread over the week [33].

Therefore, to provide more evidence on the relationship between alcohol and cancer, we aimed to evaluate the association between the MADP and alcohol-related cancers in the SUN (“Seguimiento Universidad de Navarra”) cohort.

## 2. Materials and Methods

### 2.1. Study Population

**Figure 1:**
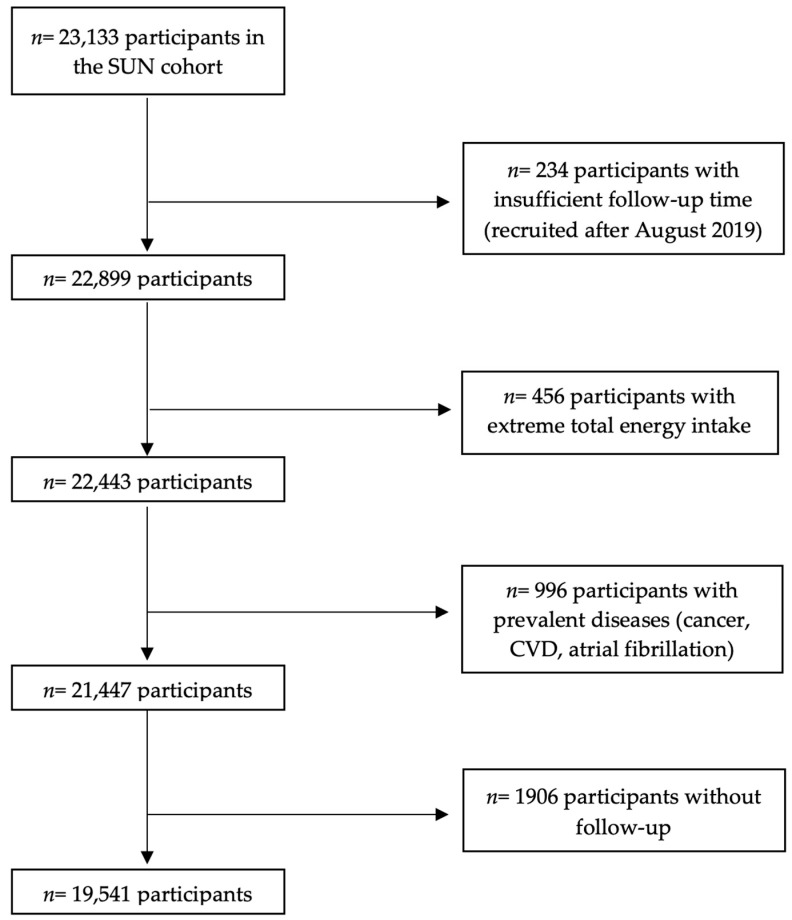
Flow chart of participants in the “Seguimiento Universidad de Navarra” (SUN) Project. SUN: Seguimiento Universidad de Navarra; CVD: Cardiovascular disease.

The SUN Project is a prospective, dynamic and multipurpose cohort of Spanish university graduates. Recruitment has been open since 1999 and is still open. Participants receive a baseline paper-based or electronic questionnaire, which inquires about diet, lifestyles and health conditions. The information is updated biennially. Further explanation on the design and methods of the SUN cohort has been previously described [34,35,36]. Figure 1 shows the selection of the analytical sample. Up to May 2022, 23,133 subjects had answered the baseline questionnaire. For the present analysis, 234 participants with insufficient follow-up time, 436 subjects with total energy intake out of percentiles 0.5 and 99.5, and 996 participants with prevalent diseases (cancer, cardiovascular disease or atrial fibrillation) were excluded. Among the remaining 21,447 subjects, 19,541 were successfully followed-up (overall retention: 91.1%). The present study was approved by the Institutional Review Board of the University of Navarra. Voluntary informed consent was obtained from all participants by completing the baseline questionnaire at no cost, using methods approved by our Institutional Review Board.

### 2.2. Mediterranean Alcohol-Drinking Pattern (MADP)

Alcohol consumption at baseline was assessed using a 136-item semi-quantitative food frequency questionnaire (FFQ) [37]. Data were also obtained on participants’ drinking habits over the year preceding the completion of the baseline questionnaire. Adherence to the MADP was assessed using the previously defined MADP score [21]. The moderate total alcohol consumption item was modified to better fit evidence and current recommendations on grams of moderate alcohol consumption. This score ranged from 0 to 9 points and assessed adherence to specific criteria, including (1) moderate total alcohol consumption: 2 points for alcohol consumption of 5–15 g/day for women and 10–30 g/day for men. Intake below this range (0–5 g/day for women and 0–10 g/day for men) scored 1 point, while intake above this range (>15 g/day for women and >30 g/day for men) received 0 points. (2) Preference for wine: 1 point was given if at least 75% of the alcohol consumed was in the form of wine. (3) Preference for red wine: 1 point was assigned if at least 75% of the wine consumed was red wine. (4) Consumption of wine with meals: 1 point was scored if at least 75% of the wine was consumed during mealtimes. (5) Low consumption of spirits: 1 point was given if the consumption of spirits accounted for less than 25% of the total alcohol consumption. (6) Alcohol consumption spread over a week: 2 points were scored for participants in the highest quartile of the ratio of number of drinking days per week to total g/week of alcohol intake. One point was scored for participants in the third and second quartiles and zero points for those in the lowest quartile. (7) Avoidance of binge drinking: 1 point was given for never exceeding five drinks on a single occasion.

These cut-off points were defined according to previous research on Mediterranean drinking patterns [21,38]. The MADP score was then collapsed into three categories: 0–3 points (low adherence), 4–6 points (moderate adherence) and 7–9 points (high adherence). Participants who reported not drinking alcohol (abstainers) were excluded from the MADP scoring and considered as an additional, separate group.

### 2.3. Outcome Assessment

The outcome involved the diagnosis of (or death from) any of the 7 cancer sites recognized by IARC as being causally related to alcohol consumption: oral cavity, pharynx, larynx, esophagus (squamous cell carcinoma), colorectum, liver (hepatocellular carcinoma) and cancer of the breast. Participants first reported their cancer diagnosis in follow-up questionnaires. A medical report was then requested to confirm their diagnoses. Confirmation of cancer cases was made by independent oncologists who were blinded to the exposure. In addition, the Spanish Statistics National Institute (“Instituto Nacional de Estadística”, INE) was consulted yearly to determine the cause of death of the study participants, and the outcome was confirmed if the cause of death was a cancer in any of the previously mentioned sites.

### 2.4. Covariate Assessment

We collected information on anthropometric and socio-demographic variables such as sex, age, height, weight and marital status [39]. The baseline questionnaire also gathered information on lifestyle variables, such as smoking habits or physical activity [40]. In addition, participants reported their medically diagnosed conditions, screening tests and medical history. Finally, adherence to the Mediterranean diet was estimated using the validated 136-item FFQ [41] to derive the Mediterranean Diet Score (MDS) [42], omitting the alcohol component to prevent overlap with our main exposure.

### 2.5. Statistical Analysis

Participants were classified into 4 groups, namely abstainers and 3 further groups according to their MADP score adherence (low, moderate or high). Baseline characteristics are shown according to these categories, separately by sex and adjusted with inverse probability weighting (IPW) for age. For categorical variables, we summarized the data as percentages, whereas for continuous variables, we reported means and standard deviations (SDs). To evaluate the association between MADP adherence categories and the incidence of alcohol-related cancers, we fitted Cox proportional hazard models. Hazard ratios (HRs) and 95% confidence intervals were computed, using the low-adherence group as the reference category. In the Cox regression models, with age as the underlying time variable, the enter time was the date of completion of the baseline questionnaire and the exit time was the date of the alcohol-related cancer diagnosis (for confirmed cases), last contact (for survivors) and the date of death (for deceased participants). Furthermore, age was the underlying time variable (birthday as origin). Multivariate models were stratified by year of recruitment (1999/2003, 2004/2009, 2010/2022) and age groups (decades). Analyses were performed separately by sex. Multivariate models were adjusted for potential confounders such as baseline body mass index (BMI, kg/m^2^; continuous), total energy intake (kcal/day; continuous), adherence to MDS (3 categories), marital status (dichotomous), physical activity (MET-h/week; tertiles), smoking habit (four groups and package-years of cumulative exposure, four categories), family history of breast cancer (dichotomous), family history of colon cancer (dichotomous), having undergone a mammography (dichotomous), having undergone a colonoscopy (dichotomous), years of university studies (years; continuous), prevalent depression (dichotomous) and chronic diseases at baseline (hypercholesterolemia, hypertriglyceridemia, diabetes, hypertension; dichotomous).

We also examined the association of a continuous 2-point increase in the MADP score with the risk of alcohol-related cancer incidence in the Cox regression models after removing abstainers.

Finally, we performed sensitivity analyses by assessing the models under several alternative scenarios: excluding breast cancer cases, using energy limits suggested by Willett, including alcohol in MDS; excluding participants if there are prevalent metabolic conditions (hypercholesterolemia, hypertriglyceridemia, diabetes, hypertension); excluding participants with cancers in first 2 years of follow-up; excluding smokers and former smokers; excluding never smokers and including only older than 50 years at baseline.

All *p* values were two-tailed, and 95% confidence intervals (95% CI) were computed. Statistical analyses were carried out using STATA/SE, version 15.1 (StataCorp, College Station, TX, USA).

## 3. Results

All the analyses were performed separately for men and women. In Table 1 we show the baseline characteristics of participants adjusted for age with inverse probability weighting. However, after adjusting for age using the IPW, there were few differences in other characteristics between the three MADP adherence groups.

During a median follow-up of 13.8 years (interquartile range: 8.8–17.3), 268 participants (75.4% women) received a confirmed diagnosis of an alcohol-related cancer. The leading cancer site was female breast cancer (149 cases) followed by colorectal cancer (70 cases). The main type of breast cancer in women was luminal (58.4%) and premenopausal cancer (56.4%). The mean age at cancer diagnosis was 54 years (SD = 11.2).

Hazard ratios (HRs) for alcohol-related cancer incidence according to the four defined groups of alcohol-drinking patterns are shown in Table 2. Different HRs were found in the stratified analysis by sex. Among men, those with the highest adherence to the MADP score (7–9 points) showed the lowest risk of alcohol-related cancer incidence in the multiple-adjusted model (HR = 0.44, 95% CI (0.21–0.92)) when compared to their counterparts with low adherence to the MADP. Furthermore, participants with a moderate adherence to the MADP showed a reduction in alcohol-related cancer incidence that tended towards statistical significance (HR = 0.56, 95% CI (0.30–1.06)).

Moreover, when we studied the MADP score as a continuous variable, we found that a two-point increment in this score was inversely associated with the risk of alcohol-related cancer incidence, but in the multivariable-adjusted model, this continuous inverse association lost its statistical significance (HR = 0.76, 95% CI (0.57–1.02)).

However, adherence to the MADP in women was not associated with incidence of alcohol-related cancers even in the high-adherence group (HR = 1.13, 95% CI (0.68–1.87)) compared to the low-MADP-adherence group. Moreover, when the MADP score was modeled as a continuous variable, a two-point increase in the score was not associated with the incidence of alcohol-related cancers (HR = 1.04, 95% CI (0.86–1.25)).

Table 3 shows the results of the sensitivity analysis for the comparison of high vs. low adherence to the MADP. A greater reduction in the risk of alcohol-related cancer incidence was observed for the only-smoker scenario in men (HR = 0.37, 95% CI (0.16–0.89)). In addition, similar reductions in the risk of alcohol-related cancer incidence were observed in men when we excluded cancers diagnosed in the first two years of follow-up (HR = 0.44, 95% CI (0.20–0.98)), when we included alcohol in the Mediterranean Diet Score (HR = 0.43, 95% CI (0.20–0.91)), and when we used Willett’s total energy intake limits instead of percentiles (HR = 0.46, 95% CI (0.22–0.95)). The remaining analyses in men showed an inverse but statistically non-significant association. In the same way, the interaction with smoking was evaluated, as different results were observed for each stratum, but it was not statistically significant (*p* = 0.717). Sensitivity analyses in women showed no scenario with statistically significant results. However, there were two analyses with inverse albeit non-significant associations: when smokers were excluded (HR = 0.73, 95% CI (0.34–1.58)) and when Willett’s total energy intake limits were used instead of percentiles (HR = 0.94, 95% CI (0.54–1.64)). We also evaluated the interaction with smoking in women and, as observed in men, it was not statistically significant (*p* = 0.121).

## 4. Discussion

We found different associations between MADP and alcohol-related cancer incidence by sex. For women, the association found between adherence to the MADP and cancer risk did not provide sufficient evidence to add to or modify current alcohol recommendations [43,44]. We did not find any inverse association between the MADP and alcohol-related cancers in women. This is consistent with previously available evidence for breast cancer, the most common cancer in women, which is linearly related to alcohol consumption, without any safe level. In fact, abstinence from alcohol is currently recommended as one of the major protecting factors for breast cancer [7,45,46]. On the other hand, the results found in men showing an inverse association between the MADP and alcohol-related cancers, suggested an additional advice for drinkers. It is not only the amount of alcohol consumed that is relevant but also the drinking pattern. In men, better adherence to the MADP was associated with a substantial reduction in the risk of alcohol-related cancers as compared to low adherence. This seems plausible, given that the average amount of alcohol intake was only 12.3 g/d (SD = 7.6) in the high category of the MADP and 17.2 g/d (SD = 24.9) in the low MADP category.

Traditionally, the effects of alcohol have only been considered in terms of the total amount consumed without taking other aspects into consideration. Moderate consumption has been associated with both beneficial and harmful effects. Inverse associations have previously been observed between adherence to the MADP and mortality [21,22], CVD [23] and diabetes [24]. However, there is no previous evidence of an association between MADP and cancer risk.

The pattern of alcohol consumption, like dietary patterns, is a better reflection of actual consumption and a more comprehensive assessment for the study of its association with events such as cancer [17,47]. However, other factors, such as sex, may influence this relationship. Current evidence generally conveys the same public health messages to both men and women, focusing on the amount of alcohol consumed and its associated cancer risks [1,7]. However, as suggested by our results, additional advice should be given to men who drink alcohol. Male drinkers with high adherence to the MADP were found to have a lower risk of alcohol-related cancers than those with low adherence to this drinking pattern. One of the possible mechanisms that could explain this protective effect is moderate consumption. Colorectal cancer, the most common alcohol-related cancer in men, is associated with high alcohol consumption [7]. The MADP penalizes this type of consumption, so that people who drink excessively or binge drink have low adherence, and therefore, high adherence to the MADP is expected to render inverse associations. Red wine is also the main alcoholic beverage in the MADP, with anti-inflammatory and antioxidant effects attributed to its high polyphenol content. The best known of these polyphenols is resveratrol, whose effects include reducing inflammation caused by reactive oxygen species and inflammatory cytokines [48]. However, resveratrol is not the only phenolic compound in red wine. Wine flavonoids also exert beneficial effects by acting as potent antioxidants, protecting against LDL-cholesterol oxidation and atherosclerosis [49]. In addition, they contribute to improved insulin sensitivity and help preserve mitochondrial function [50,51,52]. These compounds have been associated with the prevention of several chronic diseases, particularly cardiovascular and neurodegenerative disorders [53]. Another item in the MADP is the consumption of alcohol with meals, which strongly influences its metabolism. Firstly, the presence of food during alcohol intake leads to a ‘washout effect’, whereby the ethanol spends less time in contact with the oropharyngeal mucosa, thus reducing its local toxicity [30]. In addition, the food delays gastric emptying and activates metabolic enzymes, increasing their activity and contact time. As a result, the liver does not saturate its enzymatic capacity and there are fewer toxic by-products, such as acetaldehyde, released into the plasma which can damage other tissues. In conclusion, by promoting moderate consumption with food, the MADP may enhance proper alcohol metabolism, which, together with the polyphenols in wine, may be responsible for the cancer protective effect seen in men. This inverse association was observed when the high-adherence group was compared with the low-adherence group.

In our study, we found that there was no statistically significant association with alcohol-related cancer incidence when comparing high to low adherence to the MADP in women, (HR = 1.13, 95% CI (0.68–1.87)), or when comparing the abstainer group versus low MADP adherence in women (HR = 1.12, 95% CI (0.70–1.79)). This may be because the main cancer in women is breast cancer (88.1%) and the available evidence suggests a linear increase in cancer risk [7,45]. Alcohol may increase circulating estrogen and reactive oxygen species, thereby inducing tumor cell proliferation [3]. These findings are consistent with previous evidence from other prospective cohorts [17,20], which reported significant associations between alcohol and breast cancer even among light consumers. There are also studies, such as a case–control study in France, that found that low to moderate consumption of regular wine did not increase the risk of breast cancer [54]. Thus, patterns of alcohol consumption may also affect cancer in women. The lack of statistically significant results may also be due to suboptimal statistical power. To confirm the evidence, the effect of MADP should be further investigated in cohorts with a higher incidence of cancer in women or in clinical trials.

Abstainers were not used as a reference group because of the possible “sick quitter hypothesis”, which holds that some abstainers avoid alcohol because they have a pre-existing medical condition. On the other hand, an inverse—although non-significant—association was observed for abstainers when compared to their counterparts with low adherence to the MADP; a protective effect could be intuitive, but it was not statistically significant (HR = 0.64, 95% CI (0.23–1.79)) among men.

After several sensitivity analyses, most scenarios showed similar results to the main analysis. In men, a greater effect was observed when only smokers were included (HR = 0.37, 95% CI (0.16–0.89)). This result led to an assessment of a possible interaction with smoking, but it was not statistically significant (*p* = 0.121). With this result, it could be concluded that men who also smoked had a greater risk reduction (63%) if they adhered to the MADP. The remaining analyses showed very similar results, all with inverse associations, although some of them lost statistical significance. In women, none of the analyses were statistically significant, and most showed similar results to the main analysis. When smokers were excluded (HR = 0.73, 95% CI (0.34–1.58)) or when the energy limits proposed by Willett were used (HR = 0.94, 95% CI (0.54–1.64)), inverse associations with cancer incidence were observed, but none of them were statistically significant. These analyses may suggest suboptimal statistical power in women when analyses were stratified by sex.

Our study has some limitations that warrant consideration. The SUN cohort consists predominantly of relatively young adults, so chronic conditions or diseases typical of older age may be less represented and affect the statistical power of the results. Secondly, as a cohort of university graduates, it is not representative of the general population, and therefore, its external validation is limited. However, the generalizability of the results must be based on biological plausibility. Thirdly, since information on alcohol consumption is self-reported, there may be some misclassification, which could affect alcohol consumption. However, previous validation sub-studies for the dietary habits’ questionnaire [37] showed a high correlation coefficient for alcohol (r = 0.88). Fourthly, for alcohol consumption, we do not have data on lifetime consumption, so we cannot distinguish between former drinkers and lifetime abstainers. However, by choosing low pattern adherence as the reference category, the problem of the ‘sick quitter’ effect is excluded. Fifth, breast cancer is the main confirmed cancer among women in our cohort, which limits the statistical power to detect associations for other alcohol-related cancers in this group. However, these figures are consistent with population-based data, where breast cancer is the most frequently diagnosed cancer worldwide [2,3]. Finally, residual confounding bias cannot be excluded.

The strengths of this study are its large sample size, high retention rate and long follow-up. In addition, this cohort has many covariates, so we have adjusted for important potential sources of confounding. Moreover, although the cancer cases are self-reported, they have all been confirmed by a trainee oncologist and medical reports. Lastly, the fact that this is a highly educated cohort reduces confounding by educational level and improves the quality of self-reported data.

## 5. Conclusions

This article highlights the importance of sex-specific messages regarding alcohol consumption and the incidence of alcohol-related cancers. For women, according to our results, we did not have sufficient evidence to add to or change the current recommendations, and the available evidence on linear increasing risk with increasing consumption from the first drink should be maintained [7,17,20]. However, for men, if they drink, it should be recommended to follow the MADP, not only because of the benefits already seen in reducing mortality, CVD and diabetes but also because of the 56% reduction in alcohol-related cancer risk that we observed. Therefore, in the absence of clinical trials providing more robust evidence, these observational results can be used and recommended in clinical practice.

## Figures and Tables

**Table 1:** Baseline characteristics of participants after adjustment for age by inverse probability weighting.

MEN	Mediterranean Alcohol-Drinking Pattern			
	Abstainers	Low (0–3)	Moderate (4–6)	High (7–9)
**N**	613	1633	3416	1433
**Alcohol intake (g/d)**	0	17.2 (24.9)	13.5 (13.5)	12.3 (7.6)
**Total energy (kcal/d)**	2498.9 (852.8)	2595.9 (826.5)	2537.8 (775.1)	2474.7 (763.9)
**Body-mass index (kg/m^2^)**	25.2 (3.4)	25.8 (3.3)	25.6 (3.2)	25.3 (2.8)
**Smoking habit**				
**Never smokers (%)**	61.1	40.3	42.5	44.3
**Former smokers (%)**	25.3	35.5	34.6	36.3
**Current smokers and <20 PackYear (%)**	8.5	17.1	17.4	15.3
**Current smokers and ≥20 PackYear (%)**	5.1	7.2	5.5	4.1
**Mediterranean Diet Score (Trichopoulou, 0 to 8)**				
**<3 points (%)**	28.1	22.7	20.2	23.3
**3–5 points (%)**	50.5	58.2	59.6	57.2
**>6 points (%)**	21.4	19.1	20.3	19.6
**Depression (%)**	10.0	9.9	9.1	10.4
**Chronic diseases (%) ^1^**	35.6	39.6	37.0	36.5
**Having undergone a colonoscopy (%)**	11.1	12.6	11.1	13.3
**Family history of colon cancer (%)**	6.0	5.7	5.7	6.4
**Family history of breast cancer (%)**	7.9	9.6	10.1	8.2
**Physical activity (MET-h/week)**				
**Tertile 1 (%)**	30.1	29.3	26.3	23.7
**Tertile 2 (%)**	32.2	32.7	32.8	34.5
**Tertile 3 (%)**	37.8	38.0	40.9	41.7
**Marital status: married (%)**	58.1	58.5	63.1	63.4
**University education (years)**	5.4 (1.8)	5.3 (1.6)	5.4 (1.7)	5.7 (1.9)
**WOMEN**	**Mediterranean Alcohol-Drinking Pattern**			
	**Abstainers**	**Low (0–3)**	**Moderate (4–6)**	**High (7–9)**
**N**	2586	2704	4432	1258
**Alcohol intake (g/d)**	0	5.2 (8.2)	6.2 (6.7)	6.2 (3.7)
**Total energy (kcal/d)**	2266.5 (598.8)	2289.8 (569.6)	2300.5 (562.7)	2317.0 (565.5)
**Body-mass index (kg/m^2^)**	22.4 (3.4)	22.3 (3.3)	22.1 (3.0)	21.9 (2.8)
**Smoking habit**				
**Never smokers (%)**	67.7	42.5	46.6	53.6
**Former smokers (%)**	18.2	28.4	27.5	30.6
**Current smokers and <20 PackYear (%)**	12.1	26.2	23.6	14.4
**Current smokers and ≥20 PackYear (%)**	2.1	2.9	2.3	1.4
**Mediterranean Diet Score (Trichopoulou, 0 to 8)**				
**<3 points (%)**	23.2	21.3	21.2	23.8
**3–5 points (%)**	58.5	60.8	58.6	56.1
**>6 points (%)**	18.3	17.9	20.2	20.1
**Depression (%)**	14.8	13.8	11.4	13.7
**Chronic diseases (%) ^1^**	18.7	17.0	17.5	20.1
**Having undergone a colonoscopy (%)**	5.4	4.2	4.6	5.2
**Having undergone a mammography (%)**	33.0	36.3	35.5	39.1
**Obstetric history**				
**Age < 25 y and nulliparous (%)**	48.77	48.54	53.61	43.70
**Age ≥ 25 y and nulliparous (%)**	3.38	4.23	4.26	4.47
**First pregnancy ≤ 25 y and <30 y (%)**	13.11	13.42	16.10	16.56
**First pregnancy ≥ 30 y (%)**	14.74	12.99	15.39	14.27
**Family history of colon cancer (%)**	3.5	4.4	4.1	3.2
**Family history of breast cancer (%)**	10.7	9.2	11.1	11.8
**Physical activity (MET-h/week)**				
**Tertile 1 (%)**	39.9	39.5	35.8	35.0
**Tertile 2 (%)**	31.8	33.2	34.9	36.2
**Tertile 3 (%)**	28.2	27.3	29.4	28.8
**Marital status: married (%)**	46.1	39.6	41.0	48.9
**University education (years)**	4.7 (1.3)	4.8 (1.3)	4.9 (1.3)	5.0 (1.5)

^1^ Chronic diseases at baseline: diabetes, hypertension, hypercholesterolemia or hypertriglyceridemia. MET: metabolic equivalents.

**Table 2:** Alcohol-related cancer incidence hazard ratios (HRs) and their confidence intervals (95% CI) according to the categories of the MADP score and for each 2-point increment.

	Mediterranean Alcohol-Drinking Pattern				Only Among Drinkers
MEN	Abstainers	Low (0–3)	Moderate (4–6)	High (7–9)	Two-Point Increment
Cases/person-years	5/8457	15/21,844	31/48,253	15/21,144	
Crude	0.60 (0.22–1.65)	1 (ref.)	0.57 (0.30–1.05)	0.41 (0.20–0.84)	0.72 (0.54–0.95)
*p*	0.318		0.071	0.015	0.022
Age adjusted model	0.59 (0.21–1.64)	1 (ref.)	0.57 (0.31–1.06)	0.41 (0.20–0.84)	0.72 (0.54–0.95)
*p*	0.312		0.075	0.015	0.022
Multiple-adjusted model, *	0.64 (0.23–1.79)	1(ref.)	0.56 (0.30–1.06)	0.44 (0.21–0.92)	0.76 (0.57–1.02)
*p*	0.394		0.073	0.029	0.064
**WOMEN**					
Cases/person-years	45/35,755	30/37,855	92/61,708	35/18,397	
Crude	1.06 (0.66–1.70)	1 (ref.)	1.18 (0.77–1.79)	1.07 (0.64–1.77)	1.03 (0.86–1.24)
*p*	0.801		0.446	0.800	0.734
Age adjusted model	1.06 (0.66–1.69)	1 (ref.)	1.18 (0.77–1.79)	1.07 (0.64–1.77)	1.03 (0.86–1.24)
*p*	0.807		0.450	0.804	0.746
Multiple-adjusted model *	1.12 (0.70–1.79)	1 (ref.)	1.19 (0.78–1.81)	1.13 (0.68–1.87)	1.04 (0.86–1.25)
*p*	0.649		0.421	0.649	0.678

* Adjusted for body mass index (kg/m^2^), total energy (kcal/day), adherence to the Mediterranean diet at baseline (three categories), smoking habit (four groups and package/year, four categories), physical activity (MET-h/week, tertiles), chronic diseases at baseline (hypercholesterolemia, hypertriglyceridemia, diabetes, hypertension), prevalent depression (dichotomous), marital status (dichotomous), having previously undergone a colonoscopy (dichotomous), having previously undergone a mammography (in women, dichotomous), familiar history of colon cancer (dichotomous), familiar history of breast cancer (dichotomous), years of university education (years).

**Table 3:** Sensitivity analyses. Association of high adherence to MADP score with alcohol-related cancer incidence under a diversity of scenarios.

MEN	Cases/Person-Years	High (7–9) vs. Low (0–3)
Main analysis	66/99,698	0.44 (0.21–0.92)
Energy limits suggested by Willett	66/94,564	0.46 (0.22–0.95)
Including alcohol in the Mediterranean Diet Score	66/99,698	0.43 (0.20–0.91)
Excluding if prevalent metabolic conditions (hypercholesterolemia, hypertriglyceridemia, diabetes, hypertension)	24/64,129	0.43 (0.12–1.49)
Excluding cancers diagnosed during the first 2 years	61/84,788	0.44 (0.20–0.98)
Excluding former and current smokers	15/44,844	0.49 (0.08–2.90)
Excluding never smokers	51/54,853	0.37 (0.16–0.89)
Including only participants > 50 years at baseline	43/25,870	0.46 (0.18–1.18)
**WOMEN**	**Cases/Person-Years**	**High (7–9) vs. Low (0–3)**
Main analysis	202/153,715	1.13 (0.68–1.87)
Excluding breast cancer	24/152,242	1.02 (0.21–4.83)
Energy limits suggested by Willett	185/139,711	0.94 (0.54–1.64)
Including alcohol in MDS	185/139,711	1.14 (0.68–1.89)
Excluding if prevalent metabolic conditions (hypercholesterolemia, hypertriglyceridemia, diabetes, hypertension)	168/127,607	1.08 (0.62–1.89)
Excluding cancers diagnosed during the first 2 years	185/129,609	1.03 (0.61–1.75)
Excluding former and current smokers	78/79,088	0.73 (0.34–1.58)
Excluding never smokers	124/74,627	1.56 (0.78–3.10)
Including only participants > 50 years at baseline	35/15,205	2.88 (0.36–23.34)

## Data Availability

We will be happy to provide access to the needed dataset (including data dictionaries), making possible the replication of the main analyses used for the present article. Due to the restrictions imposed by the Informed Consent and the Institutional Review Board, bona fide investigators interested in analyzing the dataset used for the present article may submit a brief proposal and statistical analysis plan to the corresponding author. Upon approval from the SUN project Steering Committee and Institutional Review Boards, the needed data will be made available to them using an onsite secure access data enclave.

## Abbreviations

The following abbreviations are used in this manuscript:

IARC	International Agency for Research on Cancer
GBD	Global Burden of Disease
NIH	National Institutes of Health
WCRF	World Cancer Research Fund
MADP	Mediterranean Alcohol-Drinking Pattern
CVD	Cardiovascular Disease
SUN	“Seguimiento Universidad de Navarra”
FFQ	Food Frequency Questionnaire
INE	“Instituto Nacional de Estadística”
MDS	Mediterranean Diet Score
IPW	Inverse Probability Weighting
SD	Standard Deviation
HR	Hazard Ratio
BMI	Body Mass Index
CI	Confidence Interval
